# Impact of gamification on green consumption behavior integrating technological awareness, motivation, enjoyment and virtual CSR

**DOI:** 10.1038/s41598-023-48835-6

**Published:** 2023-12-08

**Authors:** Muhammad Farrukh Shahzad, Shuo Xu, Obaid ul Rehman, Iqra Javed

**Affiliations:** 1 College of Economics and Management, Beijing University of Technology, Beijing, 100124 People’s Republic of China; 2 Institute of Business and Management, University of Engineering and Technology, Lahore, 54000 Pakistan

**Keywords:** Environmental social sciences, Environmental sciences, Environmental impact

## Abstract

Gamification entails integrating game design elements, including rewards, points, competition, and interactive challenges, into non-game contexts to engage and motivate individuals. In the context of green consumption, gamification can encourage individuals to acquire more sustainable consumption behaviors. The proposed study aims to examine the influence of gamification on green consumption behavior among Chinese university students. However, university students are considered an important target group for such interventions due to their technological savvy and high interest in environmental issues. A self-determination theory (SDT) was used to measure the motivating factors of gamification for adopting green consumption behavior—a convenience sampling technique in which survey-based research designs were used to collect the data. A survey was conducted on a sample of 332 university students in China, using a questionnaire with structural equation modeling (SEM) to test the hypotheses and assess the proposed relationships between the variables. The finding of this study reveals that gamification has a significant negative relation with green consumption behavior. Further, technological awareness, hedonic motivation, and perceived enjoyment significantly mediate the relationship between gamification and green consumption behavior. Additionally, virtual CSR significantly moderates the relationship between gamification and technological awareness, hedonic motivation, and perceived enjoyment. The findings of this study could have implications for the development of more effective interventions for policy makers and industrialists aimed at promoting sustainable consumption behaviors in China.

## Introduction

With the ongoing economic growth, issues related to high carbon emissions and environmental damage have become more prominent and urgent. Due to this reason, China has planned to vigorously promote carbon–neutral techniques and introduce user-friendly policies to encourage green-consumption behaviors in society^1^. According to the latest research, green behavior is mostly influenced by the consumer's perception of consumption decisions^2^. Still, there is a need to prospect the features that help to increase green consumption behavior. These factors are mostly explored through technology and different mobile apps that trigger the effects of green consumption behavior among individuals. In addition to the extensive recognition and acceptability of video games, gamification is considered an effective strategy to engage consumers and motivate them to buy the products over a long period^3^. However, gamification is expressed as incorporating game elements into non-game contexts, which is considered an eye-catching phenomenon in the current era due to technological advancement and e-learning behavior^4^. Recent research scholars have highlighted that it is worthwhile for companies to integrate game elements into non-gaming contexts such as commercial activities^5^. It would ultimately affect the user's purchase decision-making process in the virtual environment.

Additionally, past scholars^6^ have revealed limited research on the context of gamification and its effect on consumer green consumption behavior; therefore, this study is conducted to highlight gamification's role in green consumption behavior with different factors. Multiple studies have been conducted on using and adopting gamification technology in e-commerce, such as Amazon, Flipkart, and Myntra, to create customer engagement and loyalty^7^. However, past researcher^8^ has explored that enjoyment plays an integral part during online shopping because young consumers, especially students, feel applauded and encouraged to experience playing games regarding gamification. It is considered the one aspect of contribution in current research. In contrast, hedonic motivation is another way to mediate the relationship between gamification and green consumer behavior, less explored in Chinese students^9^. However, hedonic motivation is pursuing pleasure and avoiding pain^10^. In the context of gamification, hedonic motivation can be used to encourage users to engage in desired behaviors or activities by making them enjoyable and rewarding. Similarly, technological awareness motivates individuals' green consumption behavior^11^. Although different studies have used the concept of technological awareness in multiple aspects, such as the adoption of gamification technology^12^, consumer intention for using the technology^13^, and consumer reaction towards online shopping^14^, in this a past research was conducted in the context of Chinese students' green consumption behavior^15^ which is considered another major contribution of the current study. However, the main purpose of the current study is to investigate the influence of gamification on green consumption behavior in Chinese students, along with multiple factors that help to promote sustainable practices in society.

According to the latest consumer survey, green consumption behavior can be enhanced by adopting virtual CSR practices, defined as a company's efforts to fulfill its social and environmental responsibilities using digital and virtual technologies^16^. However, in the current study, virtual CSR is used as a moderator in the relationship between technological awareness, hedonic motivation, and perceived enjoyment of the green consumption behavior of Chinese students^17^. Based on the above gaps, the current study has chosen Chinese students as a context to check the integration of gamification with green consumption behavior, technology awareness, hedonic motivation, and perceived enjoyment. The current study needs to address the issue related to the green consumption behavior of Chinese students and how gamification and multiple factors such as hedonic motivation, technological awareness, and perceived enjoyment can be tailored to the cultural and behavioral context of China to promote sustainable choices and reduce environmental impact in the society. This study contributes to the body of literature in multiple ways: first, this is the only study that measured the effect of gamification elements on green consumption behavior in the Chinese context. Second, it is considered a multi-disciplinary study as it contributes to the literature on information technology, environmental system and, sustainability and consumer behavior. Third, the virtual CSR (corporate social responsibility) is used as a moderator to intensify the relationship of gamification with other factors: technological awareness, hedonic motivation, and perceived enjoyment. Moreover, this study practically supports top management's design of organizational strategies that promote a sustainable and eco-friendly environment. They should focus on online virtual seminars, campaigns, and philanthropies to motivate the employees based on gamification reward system to encourage green consumption behavior in society.

## Theoretical foundation and hypothesis development

### Self-determination theory

Self-determination theory (SDT) focuses on human motivation and understands the factors that help to drive behavior. Edward Deci and Richard Ryan gave the concept of SDT theory, which states that Individuals have natural psychological needs that create intrinsic drive, personal well-being, and optimal functioning^18^. However, in the context of gamification and green consumption behavior, SDT provides a valuable contribution, as it directs how gamification elements affect the motivation level of individuals and consequently affect their sustainable preferences. According to this theory, humans have three basic psychological needs: autonomy, competence and relatedness; when all of them are fulfilled, it develops the behavior and motivates them to perform the task^19^. Regarding green consumption behavior, it is clarified that when Chinese students find the gamification elements such as rewards, batches, and coupons to perform any sustainable practices are enjoyable, they feel internally motivated to perform the sustainable green practices. Gamification elements provides the autonomy to the Chinese students as they are well aware of the technology and feel enjoyment, match with their value and leisure; ultimately adopts the green consumption behavior. Chinese students feel more competent; when they are well aware of the gamified technology usage and its related benefits to make the eco-friendly environment. Beside this, the hedonic motivation and virtual CSR practices enhance the sense of relatedness (caring for others) to connect the community for green consumption practices. Conclusively, this theory helps to explore how the gamification can either support or delay the individual motivation for the green consumption behavior. It also provides deeper knowledge about how gamification elements promote sustainable green consumption behavior following SDT principles, as our study aligned with past studies^20–22^.

### Gamification and green consumption behavior

Gamification applies game-like mechanics to boost motivation and involvement in marketing or education^18^. One area where gamification has been applied is in promoting green consumption behavior, which refers to individuals' choices and actions to reduce their environmental impact through sustainable consumption^23^. Gamification can encourage and reinforce green consumption behavior by making sustainable choices more fun, rewarding, and social. A company may create a mobile app that tracks an individual's carbon footprint and provides incentives for reducing it, such as points that can be redeemed for rewards. Moreover, gamified technology creates a loyalty program that rewards customers for making eco-friendly purchases, such as using a reusable coffee cup or buying organic produce^24^. Gamification is used to inform people about how their consumption affects their environmental choices, making them more aware of the consequences of their actions^25^. Games simulate the impact of different energy sources on the environment, helping individuals understand the benefits of renewable energy and the drawbacks of fossil fuels. Overall, gamification can increase engagement and motivation for green consumption behavior, leading to more sustainable choices and reducing environmental impact^26^.

However, it is important to ensure that gamification does not overshadow the underlying environmental goals and values and is used ethically and transparently^27^—one area where gamification has successfully promoted green consumption behavior among young people. Gamification has increased awareness of environmental issues among young people^28^. Through interactive games and challenges, young people can learn about the impact of their daily choices on the environment. This increased awareness can lead to greater responsibility and motivation to change consumption behavior^29^. Gamification can create a sense of community and social connection among young people. By participating in green consumption challenges and games, young people can connect with others who share their values and goals^30^. It also creates a sense of competition, which can motivate some individuals^31^. Young generations are more likely to engage in activities that they find enjoyable and entertaining. By incorporating game elements into sustainability initiatives, young people are more likely to participate and remain engaged in the long term^32^. These games can lead to lasting changes in behavior and a greater sense of responsibility for the environment^33^. As China faces a serious environmental issue, gamification appears to be a viable method for engaging youth in sustainable practices and shaping future interventions that encourage eco-friendly behavior in the younger generation^10^. From the above discussion, we propose that:

#### H_1_

Gamification will influence the green consumption behavior in university students.

### Technological awareness relation with gamification and green consumption behavior

Gamification is the usage of game design components like points, badges, and leaderboards in situations other than games to engage and motivate users^13^. By incorporating gamification elements into educational or technology-related activities, young individuals are more prone to engage with the content and develop an interest in the subject matter^4^. Gamification helps them to become more aware of different technologies and their applications. Prior studies show that gamification can also make learning about technology more interactive and fun-loving, improving retention rates and encouraging young people to continue learning about technology^34^. Furthermore, literature suggests that gamification can effectively increase technological awareness in young people and make learning about technology more engaging and enjoyable. By making the learning process more interactive and enjoyable, young people are more likely to remember what they have learned and continue seeking new information about technology^35^. Awareness of technology helps to create a positive feedback loop where increased engagement leads to increased learning, and increased learning leads to increased engagement. Prior scholars^36,37^ argued that technology awareness helps create a positive feedback loop where increased engagement leads to increased learning and vice versa. However, the prior study results show that gamification can also improve retention rates, introduce young people to new technologies, foster wisdom of competition and achievement, and create a sense of community and collaboration among young people interested in technology^38^. Therefore, gamification has the potential to be an effective tool for building technology awareness and literacy in young people and encouraging them to consider careers in technology-related fields. Based on the discussion, we propose that:

#### H_2a_

Gamification positively impacts technological awareness.

Technological awareness can also drive green consumption behavior through eco-friendly products. As people become well-informed about the environmental impact of their products, they are more inclined to choose products created from sustainable and biodegradable materials^39^. Prior literature suggested that when people become more informed about the environmental impact of their actions, they are more likely to make choices that align with their values and help protect the environment^40^. The importance of sustainable and environmentally friendly practices has become increasingly evident in past studies, particularly in the wake of climate change and other environmental challenges. Technological awareness has emerged as a key driver of green consumption behavior among young people. Multiple studies are available that used the variable of technology awareness^1,41^, but a dearth of research is available in the context of green consumption behavior^42^. Moreover, it is argued from the prior literature that many young people use technology to educate themselves about the environmental effects and make more informed choices about the products and services they consume^18^. Technological awareness positively influences green consumption behavior by providing access to information about the environmental impact of different products and services^43^. Through social media, news websites, and online forums, young people can learn about the environmental impression of products and services and their actions to reduce their carbon footprint^20^. Technological awareness plays a major role in shaping green consumption behavior among Chinese students by providing access to information, creating a sense of community, and offering tools for tracking. Technological awareness measures environmental impact and empowers young people to be more informed and sustainable. Based on discussions, we propose that:

#### H_2b_

Technological awareness positively impacts green consumption behavior.

Gamification involves using game mechanics to engage and motivate people to achieve desired outcomes. In green consumption, gamification encourages individuals to adopt environmentally friendly behaviors, such as recycling, conserving energy, and using sustainable products^44^. However, the effectiveness of gamification in promoting green consumption behavior depends on several factors, including technological awareness. Technological awareness refers to an individual's knowledge and familiarity with technology and digital platforms^12^. It includes using technology, accessing digital information, and communicating effectively online. With the help of technological awareness, gamification relies on digital platforms to deliver the game mechanics and engage users^45^. However, it is assessed that the effectiveness of gamification in promoting green consumption behavior depends on the users' technological awareness, which is less discussed in the prior studies^46^. In line with the above discussion, it is argued that individuals with high technological awareness are more likely to engage with gamification and adopt environmentally friendly behaviors. Scholars suggested that individuals with high technological awareness are more likely to be comfortable with digital platforms and technology-based interventions, such as gamification^47^. They are also more likely to be motivated to participate in such interventions and engage with the game mechanics. Technological awareness can facilitate communication and social interaction among individuals participating in gamification interventions^48^. Therefore, it is inferred that green consumption behavior would be promoted if Chinese students were aware of gamified technology usage and its environmental impact. The following hypothesis was derived:

#### H_2c_

Technological awareness mediates the relationship between gamification and green consumption behavior.

### Hedonic motivation relation to gamification and green consumption behavior

Gamification uses game design features in non-game techniques, such as education environments, to participate and motivate people to complete tasks or achieve goals. By incorporating game elements such as rewards, challenges, and social interactions, gamification can make activities more enjoyable and engaging, thus increasing hedonic motivation^33^. Past studies^49^ have shown that gamification can increase engagement, motivation, and enjoyment in various contexts, including education, health and fitness, and workplace productivity. Therefore, gamification can enhance hedonic motivation in many different areas of life. Motivation is the drive to pursue pleasure and enjoyment, and gamification uses game-like elements in non-game frameworks to engage and motivate individuals^50^. By incorporating gamification into various activities, such as learning, exercise, or work, young people can feel more motivated and engaged, leading to greater enjoyment and satisfaction. Past research^51^ has shown that gamification provides immediate feedback and rewards, which can trigger feelings of pleasure and satisfaction. Additionally, gamification can make tasks more enjoyable by adding elements of challenge, competition, and social interaction, making the task more engaging and stimulating. Past studies supported our arguments that gamification positively impacts hedonic motivation in young generations by making activities more engaging, enjoyable, and rewarding^52^. However, in the Chinese context, it is inferred from the above literature that gamification improves the excitement, joy, and source of perceived pleasure. Based on discussions, we propose that:

#### H_3a_

Gamification positively impacts hedonic motivation.

Hedonic motivation is defined as the desire to seek pleasure and avoid pain. Green consumption behavior refers to the conscious effort to purchase and use products and services that are environmentally friendly^53^. Consumers who experience positive emotions when using environmentally friendly products are likelier to continue using them. Users who feel good about using a reusable water bottle instead of a disposable one may be likelier to continue using the reusable bottle. This shows that hedonic motivation can be a powerful tool for encouraging green consumption behavior^54^. Marketers and policymakers can leverage this motivation by emphasizing the positive emotions associated with environmentally friendly products and services and highlighting the social benefits of green behavior^55^. Another way that hedonic motivation can positively impact green consumption is by encouraging young people to participate in environmentally friendly activities. These activities can be rewarding in and of themselves, as they provide a sense of accomplishment and contribute to a larger goal of protecting the environment^19^. Furthermore, scholars highlighted that hedonic motivation can be a source to encourage young people to engage in sustainable social behaviors, such as participating in community clean-up events or attending environmental rallies^34^. These activities can provide a sense of belonging and social connection, allowing young people to connect with others who share their values and environmental concerns^56^. Hence, it is argued from the above literature that if Chinese students feel gratitude and pleasure with the usage of green products, they ultimately decide to choose green consumption behavior. From the above discussion, we propose that:

#### H_3b_

Hedonic motivation will influence the green consumption behavior.

Gamification promotes green consumption by providing a fun and engaging way to learn about environmental issues and encouraging individuals to adopt pro-environmental behaviors^57^. Moreover, gamification can help to overcome barriers to green consumption behavior, such as lack of knowledge and awareness, high costs, and inconvenience. Hedonic motivation can influence individuals' engagement in gamified activities and subsequent green consumption behavior^58^. Hedonic motivation is related to the pleasure and enjoyment individuals derive from participating in a gamified activity. It can enhance individuals' intrinsic motivation to engage in pro-environmental behavior and sustain their engagement over time^59^. In a prior study, participants highly motivated by the enjoyment and excitement of the game were more likely to engage in sustainable behaviors, such as lower energy consumption and public transportation^20^. Similarly, a study by^9^ showed that hedonic motivation partially mediated the relationship between gamification and green consumer behavior in the real world. In this study, Chinese students who participated in a gamified eco-driving program reported higher levels of hedonic motivation, which, in turn, led to greater intention to adopt eco-driving behavior. The authors^60^ suggested that hedonic motivation can enhance the benefits and enjoyment of eco-driving, increasing individuals' willingness to overcome the perceived costs and barriers of adopting this behavior. Therefore, it is important to design gamified interventions that consider young people's hedonic needs and preferences, as this can increase the effectiveness and appeal of these interventions. From the above discussion, we propose that:

#### H_3c_

Hedonic motivation mediates the relationship between gamification and green consumption behavior.

### Perceived enjoyment relation with gamification and green consumption behavior

Gamification mentions using game design elements, mechanics, and principles in situations outside of games to inspire and engage people in different activities. Perceived enjoyment is a psychological construct that refers to individuals' subjective experience of pleasure and enjoyment when engaging in an activity^61^. A past study^18^ has shown that gamification can enhance perceived enjoyment in various contexts, such as education, health, and fitness. Gamification can effectively raise perceived enjoyment in various contexts by making activities fun, engaging, and motivating^5^. A past study by^56^ found that gamification elements in a fitness app increased users' perceived enjoyment and motivation to exercise. Similarly, a study by^62^ found that using gamification in an educational setting increased students' perceived enjoyment and participation in the educational process. In young generations, gamification has been used successfully in health and fitness applications to motivate young people to exercise and eat healthily. A study by^15^ found a gamified app that tracked physical activity and provided rewards for achieving goals for youth. Gamification increased students' motivation and enjoyment in a psychology course to support our study arguments^63^. Overall, gamification has the potential to impact perceived enjoyment in Chinese students positively, and it is an effective way to motivate and engage them in various contexts. From the above discussion, we propose that:

#### H_4a_

Gamification positively impacts perceived enjoyment.

Perceived enjoyment can play a prominent role in determining an individual's green consumption behavior. When individuals enjoy engaging in environmentally friendly activities, they are more likely to adopt sustainable behaviors and make greener choices in their daily lives^64^. A past study^32^ showed that individuals who perceive environmentally friendly products are more likely to recycle, reduce waste, and conserve energy. A study by^3^ found that people who enjoyed recycling were likelier to engage in recycling behavior. Additionally, our study has shown that incorporating enjoyable elements into sustainable behaviors can increase their adoption. Gamification of sustainable behavior can make it more engaging and appealing, increasing the likelihood of people adopting it^65^. Perceived enjoyment of green consumption behaviors can significantly influence an individual's likelihood of engaging in sustainable behaviors. When young people perceive environmentally friendly behaviors as enjoyable, they are more likely to engage in them^1^. Past studies argued that perceived enjoyment could be a powerful motivator for sustainable behavior^26^. According to the Chinese context, youth tend to be more environmentally conscious and aware of how their actions affect the environment. By incorporating elements of enjoyment and fun into sustainable behaviors, people may be more likely to adopt and sustain these behaviors over time^25^. Hence, from the above literature, perceived enjoyment can play a crucial role in encouraging green consumption behavior in Chinese students and can effectively promote sustainable practices. So, we propose that:

#### H_4b_

Perceived enjoyment positively impacts green consumption behavior.

In recent years, gamification has been increasingly applied to promote sustainable behaviors, such as green consumption. Green consumption refers to purchasing and using environmentally friendly products and services^19^. In gamification, perceived enjoyment may be enhanced through game design elements, such as challenges, rewards, and feedback, creating an intellect of fun, pleasure, and accomplishment^66^. The use of gamification to promote sustainable behaviors has been increasingly popular, especially among young people, who are more likely to be receptive to digital and interactive solutions. Green consumption behavior, including eco-friendly products and reducing energy consumption, is essential for addressing environmental challenges like climate change and resource depletion^67^. A past study^15^ discussed how perceived enjoyment mediates the relationship between gamification and green consumption behavior in young people. Another study^68^ showed that a gamified app, which used rewards and feedback to motivate young people to reduce energy consumption at home, resulted in significant energy savings over six months. Similarly, gamification is an educational program that uses game design elements such as challenges and points to teach young people about sustainable behaviors, increasing their knowledge and intention to adopt green consumption practices^69^. Young people, especially students, were more likely to engage in sustainable behaviors when they perceived them as enjoyable and fulfilling. Additionally, a past study^22^ investigated the enduring consequences of gamification on sustainable behaviors, and the potential unintended consequences, such as the displacement of other pro-environmental actions, are supported by our study arguments. Although perceived enjoyment has been directly discussed in multiple contexts, such as gamification, motivation, and consumption behavior, its mediating role has not yet been explored^70^. Based on literature arguments, it is concluded that Chinese students would be willing to adopt green consumption behavior if they perceived enjoyment and pleasure from using gamification elements. Hence, we hypothesized that:

#### H_4c_

Perceived enjoyment mediates the relationship between gamification and green consumption behavior.

### Moderating role of virtual CSR

Virtual CSR (corporate social responsibility) implements CSR initiatives and programs virtually or online^17^. It is a newly introduced concept in the literature, focusing on the virtual aspect of corporate social responsibility. The virtual CSR approach enables companies to engage in socially responsible activities, such as charitable donations, volunteering, and sustainability efforts, while leveraging digital tools to connect with stakeholders and deliver impact^4^. Virtual CSR can take many forms, including online fundraising campaigns, virtual events, and workshops on social and environmental issues. However, it is important to ensure that virtual CSR initiatives align with the company's values and objectives and are executed transparently and ethically^15^. With the rise of technology, virtual CSR has emerged as a way for companies to engage with customers and stakeholders in a virtual environment^71^. Technological awareness refers to an individual's knowledge and understanding of technology and its application toward environmentally friendly products and services. With the help of online platforms, such as social media and online communities, to promote virtual CSR initiatives and engage with customers^72^. Recent research^4^ has shown that virtual CSR can positively influence consumer behavior, especially among young generations who are highly connected online. A study by^73^ found that virtual CSR can increase the readiness of consumers to pay more for goods and services that are ecologically friendly. According to the SDT theory, organizations are more likely to be involved in virtual CSR efforts when they perceive autonomy in their choices, competence in understanding and participating in CSR activities, and alignment with their intrinsic values^74^. Prior literature argued that the benefits of virtual CSR include increased accessibility and reach, lower costs and reduced environmental impact, and the ability to leverage technology to measure and track impact^75^.

In addition, the previous literature suggested that hedonic motivation influenced consumer behavior through virtual CSR^76^. Virtual CSR initiatives can increase consumers' awareness and knowledge of companies' environmental and social initiatives, reinforcing their environmental attitudes and behaviors^77^. A past study^78^ highlighted virtual CSR initiatives that entertained participants through gamification. If it's catchy, it is attractive. In enjoyment, various tactics are obliged to create enhancements^79^. These enhancements are produced by featuring gamification in applications, which creates ease of use and provides enjoyment while accomplishing tasks^62^. Virtual CSR initiatives can create a sense of community among consumers, encouraging them to engage in environmentally friendly behaviors and influencing their peers to do the same^80^. Our study highlights virtual CSR initiatives' importance in enhancing green consumption behavior. The past study^81^ endorsed our arguments that companies should prioritize virtual CSR initiatives to engage with young consumers who are highly connected to online platforms, get motivated, and enjoy using technology to develop their environmental and social initiatives. Although virtual CSR has been discussed in various ways, its contribution as a moderator with technological awareness, hedonic motivation, and perceived enjoyment collectively has not yet been discussed in prior studies^27^. Hence, it is inferred from the literature that virtual CSR initiatives can create a sense of community among Chinese students and encourage them to participate proactively, which ultimately reinforces them to adopt environmental attitudes and behaviors^82^. Based on discussions, we propose that: Based on discussions, we propose that:

#### H_5a_

Virtual CSR positively moderates the relationship between gamification and technological awareness.

#### H_5b_

Virtual CSR positively moderates the relationship between gamification and hedonic motivation.

#### H_5c_

Virtual CSR positively moderates the relationship between gamification and perceived enjoyment.

**Figure 1:**
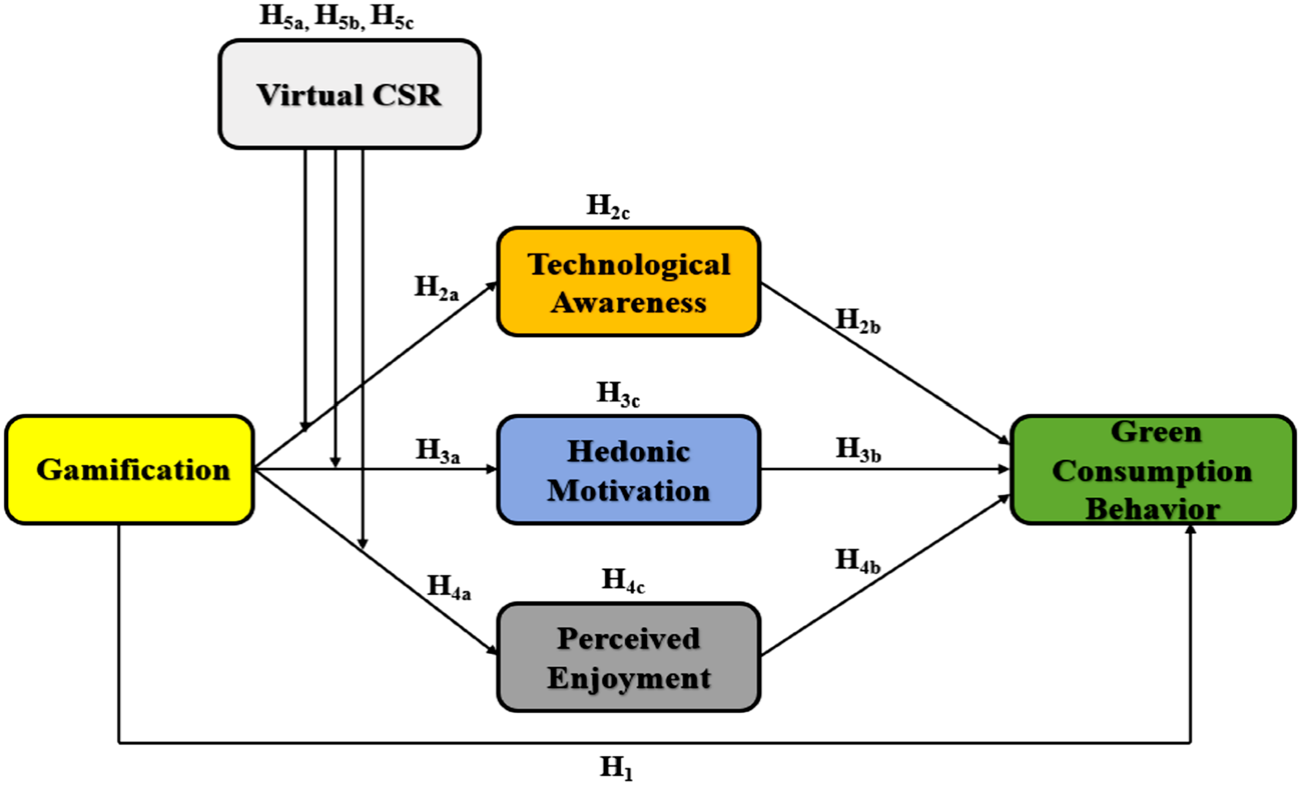
Conceptual framework.

Figure 1 explains the research model below, which depicts the literature review mentioned above.

## Methodology

### Measure and methods

Gamification has activated market-interfering devices to satisfy the needs of consumers worldwide, allowing businesses to engage with their clients and establish trends that have a significant impact^45^. In the context of Chinese students, diverse game mechanics, such as badges, levels, leaderboards, progress bars, virtual currency or tokens, awards, trading & giving, and user-to-user challenges, evolve to draw users. This paradigm empowers user instincts for gamification in human–computer interaction. Gamification fosters active user participation, enabling users to receive prizes, find flaws, and exchange ideas via feedback, opening the way for improvement. Gamification is built with perception to keep users enjoying, aware, and motivated^21^. In this study, the population sample is Chinese university students who are often considered highly educated and well aware of technology adoption and multiple usages. Hence, the data were collected from students across the top six universities in Beijing, China, including undergraduates, postgraduates, and researcher scholars. Data were collected from university students through online questionnaires from February to March 2023. Online surveys have become popular due to their convenience and accessibility, particularly among the younger generation in China^83^. For online data collection, Wenjuanxing and sojump platforms in China's most commonly used^84^. Students who use mobile technology but are less aware of gamification techniques to enhance green consumption behavior are excluded from the current study.

A total of 430 questionnaires were distributed, and 359 completed questionnaires were returned. All questionnaires returned underwent rigorous screening for missing values, multivariate outliers, and unengaged responses. Subsequently, 27 cases were discarded, leaving 332 usable cases for analysis—equating to an 83% usable response rate. According to Roscoe's (1975), a sample size greater than 30 and less than 500 is most suitable for behavioral and marketing research^85^. So, we have chosen a sample size within a range of 30–500 to get the authenticity of the current study. The researcher briefed the university students about the research agenda and guaranteed the maintenance of anonymity of personal information. A convenient sampling technique was used as an effective medium to get the appropriate results with ease to access the respondents^86^. Despite the less generalizability of results convenience sampling method is used due to easy access to the respondents and its relevance to the measurement item^4^. Hence, convenience sampling is not a major concern for our study. According to the demographic survey, female students (56%) are more inclined towards the usage of online shopping platforms than male members (44%). This study is free from common method bias as we have applied the CFA (confirmatory factor analysis) techniques to minimize the bias effect and also collected the data from multiple sources such as Wenjuanxing and Josump platforms. Similarly, the details of other demographic factors are shown below in Table 1.

**Table 1:** Demographic details of respondents.

Demographics category	(n = 332)	
	Frequency	Percentage%
Gender		
Male	146	44
Female	186	56
Age		
18–25 years old	206	62
26–30 years old	92	28
30–40 years old	34	10
Education background		
Bachelor degree	161	48
Master degree	114	34
Ph.D	42	13
Others	15	5
Daily internet use		
1–2 h	–	–
3–4 h	41	12
5–6 h	175	53
More than 7 h	116	35

### Measurements of items

Further, the current study was assessed through a self-administered questionnaire that shows the relationship among variables. For this purpose, the questionnaire is divided into two sections: the first part represents the demographic information, including age, gender, and qualification, described in Table 1. At the same time, the second part of the questionnaire is based on constructs adopted from existing studies. To measure the gamification (GAM) variable, nine items were adapted from a prior study^87^ and modified to the Chinese university students' context. However, the term GAM is defined as a technique that uses game design elements in non-game contexts to engage and motivate individuals to achieve specific goals. Similarly, we measured the Green consumption behavior (GCB) with five items derived from^1^ and adapted with minor modifications from the online service sector to the Chinese university student context. It is defined as making or purchasing environmentally friendly products^88^. To measure hedonic motivation (HMOT), five items were adapted from^89^ to the Chinese university students' context, which is defined as the desire to seek pleasure and avoid pain^10^. Moreover, a six-item scale from^13^ was adopted to measure technological awareness (TAW) from the same Chinese student context. However, the term is defined as an individual's knowledge and familiarity with technology and digital platforms^90^. To measure perceived enjoyment (PENJ), three items were adapted from^56^ and slightly changed to the current Chinese students' context to get more appropriate responses. PENJ is also defined as the degree to which someone feels or perceives enjoyment with technology. Lastly, virtual CSR's four items were measured by^70^, and minor changes were made in the context of scale. It is also defined as the use of virtual platforms such as social media and online communities to promote CSR initiatives and engage with customers^91^. All the above variables are measured on a Likert scale ranging from 1 = Highly Disagree, 2 = Disagree, 3 = Neutral, 4 = Agree, and 5 = Highly Agree. Likewise, the demographic information is assessed on a dichotomous scale such as 1 = yes, 2 = No. Details are provided in appendix A.

### Ethics statements and declarations

We confirm that all methods were carried out in accordance with relevant guidelines and regulations. Humans who participated in this study are aware of the purpose of the study, and their confidential information is not to be shared with anyone. All study participants provided their written informed consent. Study data is used after the consent of participants. The questionnaire used in this study started with the declaration and purpose of the study. Experimental protocol was approved by the ethical review board of the Beijing university of technology.

## Analysis and Results

The current study used partial least squares structural equation modeling (PLS-SEM) to measure the theoretical framework in two aspects i.e., measurement model and structural model, with the help of Smart-PLS 4.0 software^92^. Confirmatory factor analysis, path analysis, regression models, and correlation structure models are the central applications of smart-PLS-SEM^93^. The Smart PLS-SEM method enables testing complicated models with multi-level effects, such as interactions between variables in other complex models and roles as mediators^94^. This study used the partial-least-squares (PLS-SEM) technique for testing and measuring the developed model based on the data from the body of scientific literature that is currently available and the discussion above. A structural model is used to show links between the components, while a multivariate statistical technique known as partial-least-squares is used to evaluate the measurement model synchronously^95^. This study employed the Smart PLS-SEM 4.0 for data screening, analysis, and following the assumptions. This study used the bootstrapping and PLS algorithm approaches to measure construct loadings, path coefficients, and weights to evaluate the significant levels.

### Measurement model

**Figure 2:**
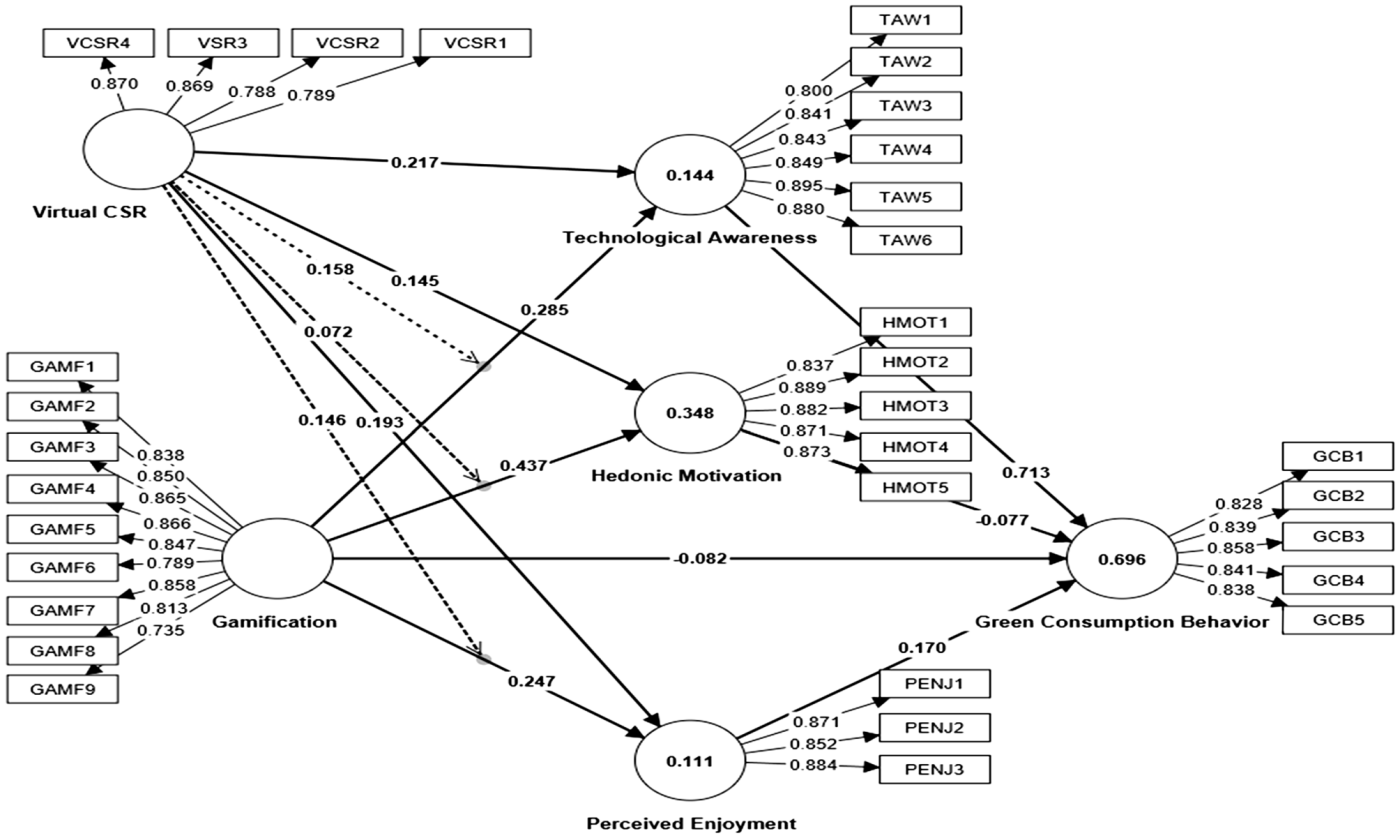
Measurement model (PLS algorithm diagram).

The measurement model tested discriminant, reliability, and convergent validity among defined constructs^94^. This study used the Smart PLS algorithm tool to determine each item's reliability and the measurement assessment of the model, as shown in Fig. 2. However, in PLS-SEM reliability of any construct is measured in terms of Cronbach's alpha, composite reliability (CR), and average variance extracted (AVE). It is represented by Cronbach's alpha (α), which shows the internal consistency among desired constructs introduced by Lee Cronbach's^96^. It shows inter-item correlation, which represents how much items of the variable are closely related to each other. High correlations among variables increase Cronbach's alpha, but a value of 1 indicates greater consistency and reliability. A Cronbach's alpha of 0.70 and above is acceptable for reliability measurement, while values above 0.70 are recommended for a good model^97^. See Fig. 2 that explains model's estimation.

Moreover, Table 2 shows that all the values of Cronbach's Alpha (α) are above the threshold of 0.70, representing the data's accuracy and reliability. Similarly, the composite reliability (CR) value is also above the significant range of 0.70, which shows the high internal consistency among all items of each variable^98^. The main aim of composite reliability is to assess each construct's reliability by measuring outer loading values in data^94^. Similarly, convergent validity is assessed using the average variance extraction (AVE) method, which measures the variance among latent constructs with a threshold value of 0.5 and excellent above 0.70^99^. In the current study, all the values of AVE are above the threshold of 0.5.

**Table 2:** Factor analysis, validity, reliability, and collinearity statistics.

Latent constructs	Items	FL	α	CR	AVE	VIF
Green consumption behavior (GCB)			0.896	0.924	0.707	
	GCB1	0.828				2.408
	GCB2	0.839				2.992
	GCB3	0.858				2.193
	GCB4	0.841				1.980
	GCB5	0.838				2.013
Hedonic motivation (HMOT)			0.920	0.940	0.758	
	HMOT1	0.837				2.488
	HMOT2	0.889				1.686
	HMOT3	0.882				2.557
	HMOT4	0.871				2.557
	HMOT5	0.873				1.867
Perceived enjoyment (PENJ)			0.838	0.903	0.756	
	PENJ1	0.871				2.706
	PENJ2	0.852				1.431
	PENJ3	0.884				2.304
Gamification (GAMF)			0.943	0.952	0.689	
	GAMF1	0.838				2.563
	GAMF2	0.850				3.214
	GAMF3	0.865				2.301
	GAMF4	0.866				1.916
	GAMF5	0.847				3.263
	GAMF6	0.789				2.743
	GAMF7	0.858				2.091
	GAMF8	0.813				1.981
	GAMF9	0.735				2.994
Technological awareness (TAW)			0.924	0.941	0.726	
	TAW1	0.800				2.950
	TAW2	0.841				3.713
	TAW3	0.843				4.053
	TAW4	0.849				2.801
	TAW5	0.895				2.741
	TAW6	0.880				1.982
Virtual CSR (VCSR)			0.850	0.898	0.689	
	VCSR1	0.789				2.325
	VCSR2	0.788				2.402
	VCSR3	0.869				2.445
	VCSR4	0.870				1.370

*FL* factor loadings, *VIF* variance inflation factor, *CR* composite reliability, *AVE* average variance extracted, *α* Cronbach’s alpha.

The concept of discriminant validity refers to the degree to which different variables in a model are distinct from one another. This is typically assessed by examining cross-loading values using two common criteria: the Fornell–Larcker criterion and the Heterotrait–Monotrait ratio (HTMT)^100^. However, HTMT is considered a superior method to Fornell–Larcker criteria because it shows clearer discrimination among constructs^101^. In the study, all the HTMT values are also within range and considered acceptable for discriminant validity. Table 3 shows discriminant validity values.

**Table 3:** Discriminant validity*.*

Constructs	GAM	GCB	HMOT	PENJ	TAW	VCSR
Fornell–Larcker criterion						
GAM	0.830					
GCB	0.134	0.841				
HMOT	0.564	0.118	0.871			
PENJ	0.260	0.777	0.225	0.869		
TAW	0.303	0.820	0.285	0.705	0.852	
VCSR	0.556	0.148	0.437	0.231	0.267	0.830
Heterotrait–monotrait ratio (HTMT)						
GAM						
GCB	0.146					
HMOT	0.602	0.129				
PENJ	0.290	0.896	0.257			
TAW	0.321	0.898	0.309	0.888		
VCSR	0.613	0.169	0.488	0.274	0.301	

*GAM* gamification, *HMOT* hedonic motivation, *PENJ* perceived enjoyment, *TAW* technological awareness, *GCB* green consumption behavior, *VCSR* virtual CSR.

### Structure model

In PLS-SEM, the structural model represents the relationships among latent variables in the conceptual model. To access direct and indirect relationships between the constructs, this study applies structural equation modeling (SEM)^102^ with partial least squares (PLS)—a more appropriate way to predict and explore structural relationships^103^ as it provides access to full and partial mediation^96^. Similarly, the variance inflation factor (VIF) is evaluated for more appropriate results, which displays the multicollinearity among constructs^104^. The acceptable range of multicollinearity is less than 5, which shows that all the variables in the study are highly correlated. The bootstrapping method was used to resample with a setting of 5000 resamples and a 95% bias correction to examine the significance of the structural path coefficients and coefficients of determination R^2^^105^.

However, Table 4 shows the result of bootstrapping in the model, which shows that all direct and indirect variables show significant relationships. Gamification negatively impacts green consumption behavior with values (β = − 0.082, t = 2.759, and p < 0.05), as supported by H1. Furthermore, gamification significantly impacts technological awareness with values (β = 0.285, t = 4.841, and p < 0.05), as validated by H2a. Technological awareness positively impacts green consumption behavior (β = 0.713, t = 11.490, and p < 0.05), as supported by H2b. Gamification significantly impacts hedonic motivation with values (β = 0.437, t = 7.536, and p < 0.05), as validated by H3a. Hedonic motivation negatively impacts green consumption behavior (β = − 0.077, t = 2.891, and p < 0.05), as supported by H3b. Gamification significantly impacts perceived enjoyment with values (β = 0.247, t = 4.203, and p < 0.05), as validated by H4a. Perceived enjoyment negatively impacts green consumption behavior (β = 0.170, t = 2.694, and p < 0.05), as supported by H4b.

**Table 4:** Hypothesis testing.

Path	β-values	Mean	STD	t-values	p-values	Results
Direct effect						
GAM → GCB (H_1_)	− 0.082	− 0.081	0.030	2.759	0.006	Accepted
GAM → TAW (H_2a_)	0.285	0.291	0.059	4.841	0.000	Accepted
TAW → GCB (H_2b_)	0.713	0.710	0.062	11.490	0.000	Accepted
GAM → HMOT (H_3a_)	0.437	0.438	0.058	7.536	0.000	Accepted
HMOT → GCB (H_3b_)	− 0.077	− 0.076	0.027	2.891	0.004	Accepted
GAM → PENJ (H_4a_)	0.247	0.253	0.059	4.203	0.000	Accepted
PENJ → GCB (H_4b_)	0.170	0.174	0.063	2.694	0.007	Accepted
Mediation effect						
GAM → TAW → GCB (H_2c_)	0.203	0.206	0.043	4.763	0.000	Accepted
GAM → HMOT → GCB (H_3c_)	0.034	0.033	0.012	2.749	0.006	Accepted
GAM → PENJ → GCB (H_4c_)	0.042	0.044	0.020	2.127	0.033	Accepted
Moderation effect						
VCSR*GAM → TAW (H_5a_)	0.158	0.165	0.060	2.625	0.009	Accepted
VCSR*GAM → HMOT (H_5b_)	0.072	0.070	0.035	2.052	0.040	Accepted
VCSR*GAM → PENJ (H_5c_)	0.146	0.151	0.060	2.436	0.015	Accepted

*GAM* gamification, *HMOT* hedonic motivation, *PENJ* perceived enjoyment, *TAW* technological awareness, *GCB* green consumption behavior, *VCSR* virtual CSR, *STD* standard deviation.

**Significant at p < 0.05.

**Figure 3:**
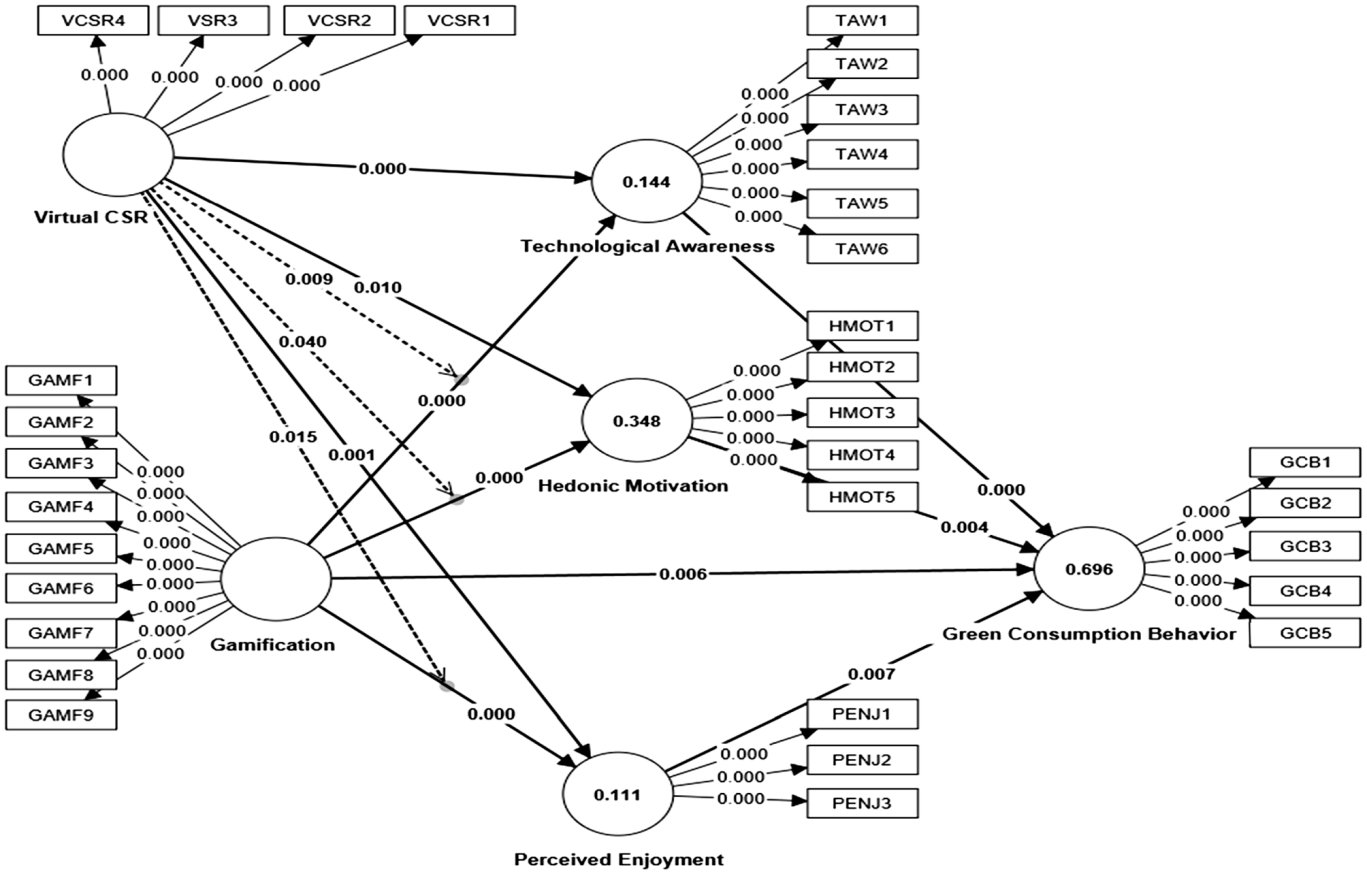
Structural model (PLS-SEM bootstrapping analysis).

For meditation analysis, our study of three mediators such as technological awareness, hedonic motivation, and perceived enjoyment, significantly mediate the relationship between gamification and green consumption behavior with values (β = 0.713, − 0.077, 0.170, t = 11.490, 2.891, 2.694 and p < 0.05), respectively. Thus, H2c, H3c, and H4c are supported. For moderation analysis, our study shows findings virtual CSR significantly moderates the relationship between technological awareness, hedonic motivation, and perceived enjoyment (β = 0.158, 0.072, 0.146, t = 2.625, 2.052, 2.436, and p < 0.05), respectively. Thus, H5a, H5b, and H5c are validated. Values of R^2^ reflect how well the IVs are able to describe the DVs^106^. The R^2^ values of this research model are green consumption behavior = 69.6%, technological awareness = 14.4%, hedonic motivation = 38.2%, and perceived enjoyment = 11.1%, respectively. All the path coefficients and values of R^2^ are shown below in Fig. 3.

**Figure 4:**
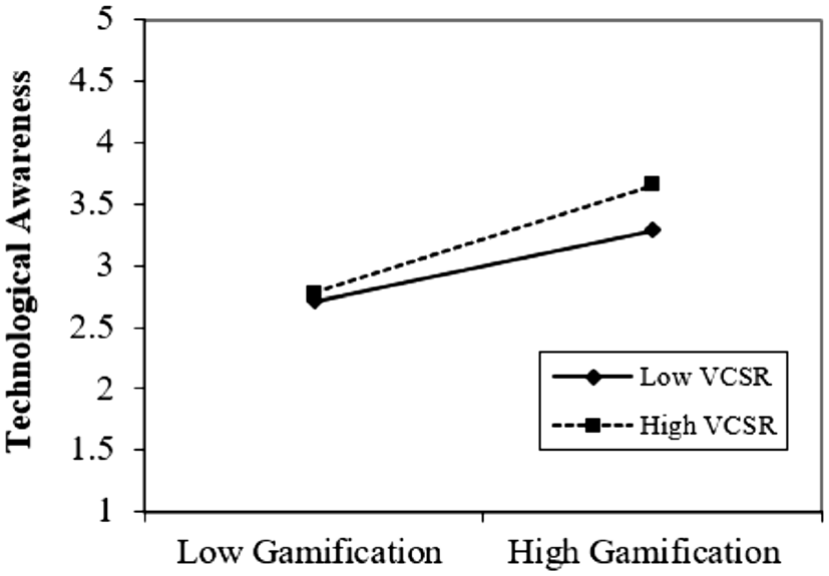
Moderating graph of VCSR between GAM and TAW.

**Figure 5:**
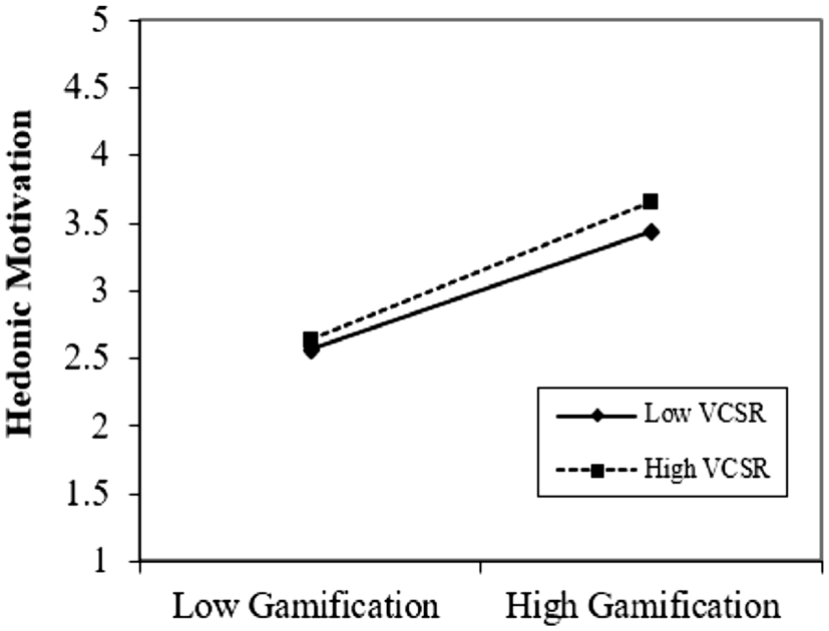
Moderating graph of VCSR between GAM and HMOT.

**Figure 6:**
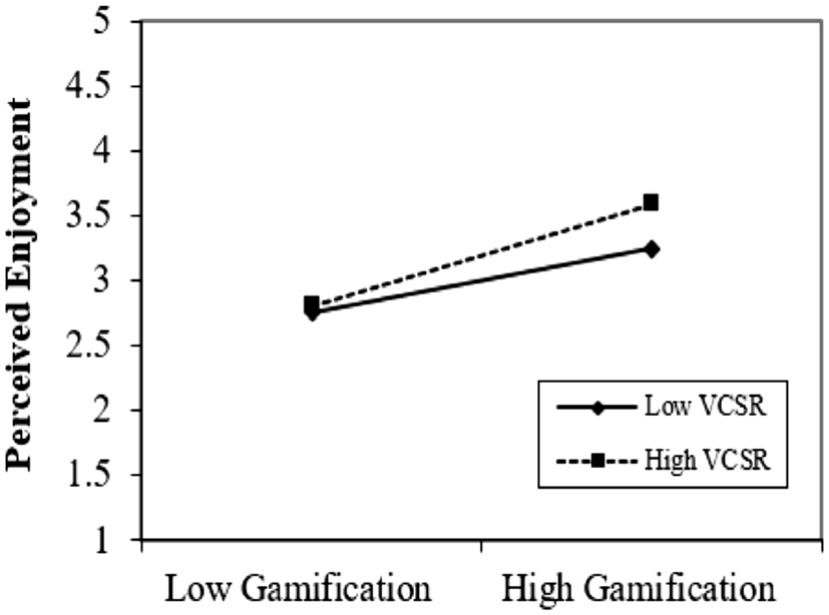
Moderating graph of VCSR between GAM and PENJ.

We illustrated the graphs to enlighten the outcomes of moderating the role of virtual CSR. Figures 4, 5 and 6 show that virtual CSR moderates the association between gamification and technological awareness, hedonic motivation, and perceived enjoyment. Figure 4, describes the moderating role of virtual CSR between gamification and technological awareness. The prominent point on the moderation graphs is where gamification and technological awareness are stronger under high virtual CSR. Figure 5, elucidates the moderating role of virtual CSR between gamification and hedonic motivation. The prominent point on the moderation graphs is where gamification and hedonic motivation are stronger under high virtual CSR. Figure 6, clarifies the moderating role of virtual CSR between gamification and perceived enjoyment. The distinguished point on the moderation graphs is where gamification and perceived enjoyment are stronger under high virtual CSR. Hence, it also supported H5a, H5b, and H5c.

## Discussion

The internet has developed into practical information in the twenty-first century, changing the traditional global system into online applications^107^. In order to increase motivation, enjoyment, and awareness, gamification involves integrating game concepts such as websites, online communities, and other online applications. Gamification aims to engage people and encourage them to cooperate with other people^108^. The main goal of the current study is to inspect the influence of gamification on green consumption behavior in the young generation of China through technological awareness, hedonic motivation, and perceived enjoyment. Additionally, it inspects the moderating role of virtual CSR between gamification and technological awareness and hedonic motivation and perceived enjoyment towards green consumption behavior. Data was gathered from 332 students who are using online platforms. The questionnaires were given to the targeted sample to get their opinion about gamification use in green consumption behavior. The results are deliberated in five major portions: first, the findings indicate that gamification significantly negatively influences green consumption behavior. Gamification can be an active strategy for encouraging green consumption behavior, as it provides users with immediate feedback, social recognition, and intrinsic motivation to engage in sustainable behaviors^109^. A study by^21^ examined the impact of gamification on household energy conservation. They developed a gamified mobile app that gave users feedback on their energy use, offered energy-saving tips, and rewarded them with virtual badges and points for reducing their consumption, supporting our study arguments. With the help of our study findings, gamification can be useful for promoting sustainable behavior with the help of technological awareness. Still, it should be designed and implemented carefully to maximize its effectiveness and avoid unintended consequences.

Second, this study investigated the mediating role of technological awareness between gamification and green consumption behavior. The results show that the relation between gamification and green consumption behavior is significantly mediated by technological awareness, which the prior literature confirms^65^. A prior study also highlighted that technological awareness referred to increasing awareness of environmental issues; there has been a growing interest in green consumption behavior, which involves making eco-friendly choices when purchasing products and services^110^. Consumers use mobile applications to scan product barcodes and obtain information about the products through technological awareness, giving individuals easy access to information about sustainable practices and products^69^. As technology advances, harnessing its power to promote a sustainable living environment and protect sustainable consumption behavior is essential.

Thirdly, this study inspected the mediating role of hedonic motivation between gamification and green consumption behavior. The results show that the relationship between gamification and green consumption behavior is significantly mediated by hedonic motivation established by the prior literature^53^. A past study^111^ highlighted that gamification involves game design and mechanics to motivate and engage people in activities. It has increasingly been used to promote sustainable behavior. Hedonic motivation and gamification created a sense of enjoyment and pleasure in engaging in green consumption behavior. A past study^46^ also expressed that gamification created a sense of achievement and competition that is pleasurable and enjoyable for individuals. Hedonic motivation and gamification can promote sustainable behavior and create a more environmentally conscious society.

Fourthly, this study investigated the mediating role of perceived enjoyment between gamification and green consumption behavior. The results show that the relation between gamification and green consumption behavior is significantly mediated by perceived enjoyment, which the prior literature confirms^21^. Past research^79^ has shown that perceived enjoyment is the extent to which individuals derive pleasure or enjoyment from a particular activity or experience. A prior study^112^ highlighted that individuals who enjoy the gamified experience are more likely to engage in sustainable behaviors. People who perceive green products and services as enjoyable are likelier to engage in such behavior. They may also be more willing to pay more for environmentally friendly goods and services^62^.

Fifth, this study considered the moderating role of virtual CSR between gamification and technological awareness, hedonic motivation, and perceived enjoyment towards green consumption behavior. The results show that the relation between technological awareness, hedonic motivation, and perceived enjoyment is significantly moderated by virtual CSR, which the prior literature confirms^17^. Past research^70^ highlighted that virtual CSR refers to companies using online platforms and digital technologies to engage with their stakeholders and demonstrate their commitment to social responsibility, including social media campaigns and virtual events. Individuals with higher technological awareness and motivation are likely to be aware of the environmental impact of their activities and be more inclined to adopt green consumption practices^72^. A past study^4^ motivated individuals to enjoy sustainable practices through digital platforms such as virtual CSR that can help reinforce positive environmental attitudes and behaviors. Virtual CSR leads to enjoyment through online campaigns to promote sustainable consumption practices that can encourage individuals to adopt green behaviors.

## Conclusion and policy recommendations

Chinese university students are encouraged to engage in sustainable behavior by using gamification. Businesses can use technical savvy, hedonic motivation, perceived joy, and virtual CSR to accomplish this goal. Businesses may increase consumer engagement and enjoyment while encouraging sustainability by integrating gamification into their marketing efforts. The current research study aims to describe the role of gamification on green consumption behavior through technological awareness, hedonic motivation, and perceived enjoyment. Additionally, this study inspects the moderating role of virtual CSR between gamification and technological awareness, hedonic motivation, and perceived enjoyment towards green consumption behavior. The research study has a five-fold contribution; first, gamification significantly negatively influences green consumption behavior. Second, technological awareness significantly mediates the relationship between gamification and green consumption behavior. Third, hedonic motivation significantly mediates the relationship between gamification and green consumption behavior. Fourth, perceived enjoyment significantly mediates the relationship between gamification and green consumption behavior. Fifth, virtual CSR significantly moderates the relationship between gamification and technological awareness, hedonic motivation, and perceived enjoyment of green consumption behavior. Young Chinese consumers are motivated to make ecologically responsible purchasing decisions using gamification; therefore, policymakers and companies should take advantage of this strategy.

In accordance with the SDT theory, this study strengthens students' intrinsic motivation by providing autonomy to use the gamification elements and get perceived enjoyment, which derives from their decision-making to opt the green consumption behavior. As per our available knowledge, it is considered the first study in relation to gamification and green consumption behavior that has focused on SDT theory to widen the theory literature elements such as autonomy, competence, and relatedness. However, this study provides new insights as a major contribution to the literature of consumer psychology and information technology with the support of SDT theory. Besides all these factors, Chinese students are motivated to make ecologically responsible purchasing decisions using gamification; therefore, policymakers and companies should take advantage of this strategy.

Encourage the integration of gamified applications and platforms that raise technological awareness about environmental issues. Policymakers should support initiatives that leverage technology to inform and educate consumers about the environmental consequences of their choices. Develop and implement policies that stimulate hedonic motivation for green consumption. Hedonic motivation promotes eco-friendly leisure activities through tax breaks or discounts for green entertainment options. Encourage businesses to incorporate enjoyable and engaging elements into sustainable practices. Policymakers should work with companies to design and market green products and services in ways that make them more appealing to consumers. Recognize the moderating role of virtual CSR in promoting green consumption behavior. Policymakers should encourage companies to invest in virtual CSR initiatives, including social and environmental commitments in the virtual space, as part of their corporate responsibility strategies. Support educational programs that teach young generations about the importance of green consumption and the role of gamification by incorporating environmental education into university curricula or providing online courses and resources for adults. By implementing these policy implications, governments and other relevant stakeholders can harness the power of gamification to influence green consumption behavior and contribute to a more sustainable future.

### Theoretical and practical implications

This study contributes to the theoretical understanding of the relationship between gamification and green consumer behavior, and it highlights the potential of gamification techniques to promote sustainable consumption practices. This finding adds to the existing literature on consumer behavior and sustainability by introducing gamification as a novel approach to encourage environmentally friendly choices. It suggests that individuals more aware of technology and its potential for promoting sustainability are more likely to engage in green consumption behaviors facilitated by gamification. This finding highlights the need for education and awareness programs that promote technological literacy in the context of sustainability. The study explores the role of hedonic motivation and perceived enjoyment as underlying mechanisms through which gamification influences green consumption behavior. Showing that individuals derive pleasure and enjoyment from engaging in gamified activities promoting sustainability sheds light on the psychological factors that drive pro-environmental behavior. This finding contributes to the understanding of the motivational aspects of sustainable consumption and provides insights into the design of gamified interventions. Moreover, in line with the SDT theory, this study supports the student’s motivation by providing autonomy and competence to use the gamification elements and get the perceived enjoyment, which ultimately enhances their decision-making to choose the eco-friendly behavior. The study introduces the concept of virtual corporate social responsibility (CSR) as a moderating factor in the relationship between gamification and green consumption behavior. It suggests that the positive impact on green consumption behavior is strengthened when gamified interventions incorporate virtual CSR elements, such as virtual rewards or recognition for sustainable actions. This finding underscores the importance of incorporating CSR practices into gamification strategies to enhance their effectiveness in promoting sustainability.

The findings of this study have practical implications for the design of gamified interventions aimed at promoting green consumption behavior. By emphasizing the role of hedonic motivation and perceived enjoyment, practitioners can focus on developing gamified experiences that are engaging, fun, and enjoyable for users. It is vital for educators, marketers, and policymakers to recognize the role of hedonic motivation in promoting sustainable behavior and to develop strategies that can leverage this motivation to encourage more sustainable consumption practices among individuals. Furthermore, incorporating virtual CSR elements into gamification strategies can enhance their effectiveness by providing additional incentives and rewards for sustainable actions. The study highlights the importance of technological awareness in facilitating green consumption behavior through gamification. Practitioners can develop educational programs and initiatives that enhance individuals' understanding of technology and its potential for promoting sustainability. These programs can target different age groups and demographics to ensure widespread technological literacy, enabling individuals to make informed choices and actively participate in gamified sustainability initiatives. Organizations implementing gamified interventions to promote green consumption should collaborate with existing CSR initiatives. Organizations implementing gamified interventions to promote green consumption should collaborate with existing CSR initiatives. By aligning their efforts, organizations can leverage the reputation and credibility of CSR programs to enhance the impact of gamification on consumer behavior. This collaboration can incorporate virtual CSR elements into gamified experiences and highlight the real-world environmental impacts of consumers' actions, reinforcing their sense of social responsibility. Organizations should focus on creating games that provide a sense of achievement, competition, and social interaction, which can increase inspiration and satisfaction among participants. In education, schools and universities developed eco-friendly mobile applications or online games encouraging users to adopt sustainable behaviors.

### Limitations and future directions

This study has several limitations that should be addressed in future research. Firstly, the study was limited to the young generation of China, and the findings may not be generalizable to other age groups or cultural contexts. Secondly, this study used self-reported data, which might have been biased toward social desirability. Future research could use objective measures of green consumption behavior to overcome this limitation. Thirdly, this study did not consider the role of other factors, such as social norms or perceived interactive control, which could influence green consumption behavior. Future research could examine the interaction between these factors and gamification in promoting sustainable consumption behavior. Fourthly, this study did not explore the long-term impact of gamification on green consumption behavior. Future research could examine the sustainability of gamification as a strategy to endorse green consumption behavior over the long term. Fifthly, this study's cross-sectional design limits the ability to establish causality or determine the direction of the relationships among the variables. Longitudinal or experimental research designs would suggest stronger causal relationships between gamification, technological awareness, hedonic motivation, perceived enjoyment, and green consumption behavior. Lastly, the study examines the direct relationships between gamification, technology awareness, hedonic motivation, perceived enjoyment, and green consumption behavior, and future research could explore potential mediating variables such as self-efficacy, environmental knowledge, and attitude that explain the underlying mechanisms through which gamification influences green consumption behavior.

### Supplementary Information

Supplementary Information.

## Data Availability

The raw data supporting the conclusions of this article will be made available by the authors, without undue reservation. Data can be obtained upon reasonable request by Co-author, Muhammad Farrukh Shahzad (farrukhshahzad207@gmail.com).
