# Supplementary material for: Impact of gamification on green consumption behavior integrating technological awareness, motivation, enjoyment and virtual CSR
Source: Sci Rep. 2023 Dec 8;13:21751. doi: 10.1038/s41598-023-48835-6 (PMC10709464; doi:10.1038/s41598-023-48835-6)
Supplement: Supplementary file 1 — Supplementary Information. [file 41598_2023_48835_MOESM1_ESM.docx]

**Appendix A: Questions measuring variables**

| **Items** | **Questions** | **Source** |
| --- | --- | --- |
| **Gamification** | | |
| GAM1 | I enjoyed studying the gamified course. | [87] |
| GAM2 | The gamified course was fun to do. |  |
| GAM3 | Gamified course activities did not hold my attention at all. |  |
| GAM4 | I would describe the gamified course activities as interesting. |  |
| GAM5 | I think I did pretty well at gamified course activities. |  |
| GAM6 | I did pretty well in the gamified course activities compared to other students. |  |
| GAM7 | I feel pretty competent after working on the gamified course tasks. |  |
| GAM8 | I believe that the gamified course was of some value to me. |  |
| GAM9 | Green gamified application has a user-friendly interface. |  |
| **Hedonic Motivation** | | |
| HMOT1 | Gamification platforms often stimulate my curiosity. | [89] |
| HMOT2 | I get a lot of fun in the course of gamification platforms. |  |
| HMOT3 | The green consumption products are entertaining. |  |
| HMOT4 | My thoughts are always aroused while using games. |  |
| HMOT5 | I always feel happy with the features using game applications. |  |
| **Perceived Enjoyment** | | |
| PENJ1 | I feel excited about banking applications containing gamification. | [56] |
| PENJ2 | It is fun to use banking applications with gamification. |  |
| PENJ3 | I find it pleasant to use banking applications featuring gamification. |  |
| **Technological Awareness** | | |
| TAW1 | Customers would like to learn more about our gamification. | [13] |
| TAW2 | Customers will pay a lot of attention to anything about the brand and purchase it. |  |
| TAW3 | Anything related to our brand will grab the attention of customers. |  |
| TAW4 | I concentrated a lot on this application. |  |
| TAW5 | Customer sharing of their experience and recalling memories will help increase awareness. |  |
| TAW6 | I have confidence and am aware of green consumption behavior. |  |
| **Green Consumption Behavior** | | |
| GCB1 | I prefer purchasing green-label products. | [1] |
| GCB2 | I am willing to consider switching to other brands for ecological reasons. |  |
| GCB3 | I am willing to pay more for a healthy product that helps protect the environment. |  |
| GCB4 | I will consider buying green products because they are less polluting. |  |
| GCB5 | I introduce the green products I use to my friends and relatives. |  |
| **Virtual CSR** | | |
| VCSR1 | I satisfied the personal experiences related to gamification with virtual CSR activities. | [70] |
| VCSR2 | My friend likes or follows virtual CSR activities. |  |
| VCSR3 | I Post or share thoughts about virtual CSR activities. |  |
| VCSR4 | I post or share photos, videos, or gifts created by individuals related to virtual CSR activities. |  |
